# On assessing trait rumination using the Ruminative Response Scale

**DOI:** 10.3389/fpsyg.2024.1368390

**Published:** 2024-06-05

**Authors:** Isabell Int-Veen, Ann-Christine Ehlis, Andreas J. Fallgatter, David Rosenbaum

**Affiliations:** Tübingen Center for Mental Health, University Hospital and Faculty of Medicine, University of Tübingen, Tübingen, Germany

**Keywords:** rumination, careless responding, psychometric analysis, ruminative response scale, invalid data

## Abstract

**Introduction:**

This study explores the stability of scores on psychometrically validated trait questionnaires over time. We illustrate potential pitfalls through a larger study that used the Ruminative Response Scale (RRS) to categorize participants prior to study inclusion into two groups based on their habitual tendency to ruminate. Surprisingly, when we re-administered the RRS at the start of an experimental session, significant score changes occurred, resulting in participants shifting between the two groups.

**Methods:**

To address this, we modified our recruitment process, aiming to reduce careless responses, including an online RRS assessment a week before the lab appointment. We analyzed the different samples prior to and after changing the recruitment procedure, as well as the total sample regarding the psychometric properties of the RRS. We also explored various indices to identify and predict score changes due to careless responding; however, only a subgroup of participants was successfully identified.

**Results:**

Our findings suggest that Mahalanobis distances are effective for identifying substantial score changes, with baseline state rumination emerging as a marginally significant predictor.

**Discussion:**

We discuss the importance of conducting manipulation checks and offer practical implications for research involving psychometrically validated trait questionnaires.

## Introduction

Whenever questionnaire data are assessed, there will be a certain percentage of invalid data. Johnson (2005) summarizes three main classes of invalid data: linguistic incompetence/misunderstanding, misrepresentation, and careless response. Linguistic incompetence or misunderstanding refers to invalid data due to insufficient basic verbal comprehension. Misrepresentation, on the other hand, refers to presenting oneself in a way that is misleading or inaccurate (e.g., “faking good” and “faking bad”). Finally, careless responding is defined as follows: “Careless responding occurs when respondents fail to read or give sufficient attention to item content, resulting in data that may not accurately reflect respondents’ true levels of the constructs being measured (Meade and Craig 2012; Ward and Meade 2018)” (Ward and Meade, 2023, p. 578). Because it is difficult to find a clear definition and unique characteristics of careless responding (e.g., invariability of responses, fast responses, inconsistency), estimates of prevalence also vary substantially across studies. A recent investigation by Jones et al. (2022), where 48 crowdsourced alcohol-related studies were analyzed regarding the occurrence of careless responses, estimated the pooled prevalence rate at ∼11.7%, which is assumed to be generalizable to other fields of research (Ward and Meade, 2023). Despite the increasing knowledge and awareness of the topic, careless responding is so far rarely investigated in psychological studies, whose scientific aim is different from the explicit investigation of careless responses. That means psychological research is not routinely screened for invalid data in general. This may be due to the lack of clear guidelines concerning effective detection and elimination (Ward and Meade, 2023).

In psychology, a trait is defined as “an enduring personality characteristic that describes or determines an individual’s behavior across a range of situations” (American Psychological Association, n.d.). Usually, traits are assessed using self-report questionnaires, where different statements are rated in terms of how participants typically think or behave. An implicit assumption of these questionnaires is that they are answered in more or less the same way regardless of the situation in which they are administered, which manifests the difference between state measures (Geiser et al., 2017).

Currently, several theories exist regarding ruminative thinking (Nolen-Hoeksema et al., 2008), each with distinct emphases and implications for temporal stability (Smith and Alloy, 2009). One of the earliest theories, known as the Response Style Theory proposed by Nolen-Hoeksema (1991), primarily focuses on depressive rumination (Papageorgiou and Wells, 2004). This theory posits that rumination is a trait-like behavior, defined as “thoughts and behaviors that center on one’s depressive symptoms and their implications” (Nolen-Hoeksema and Morrow, 1991, p. 569). Nolen-Hoeksema and Morrow (1991) also introduced the Ruminative Response Scale (RRS), which remains widely used for assessing trait rumination to this day. Rumination is nowadays regarded as a transdiagnostic process apparent in many psychopathologies, which manifests the importance and relevance of investigation in the context of improving treatment options for mental disorders.

Using the data from a recent study by our group investigating the neural correlates of ruminative thinking in response to a social stress induction using the Trier Social Stress Test (TSST) (Kirschbaum et al., 1993) and the impact of Theta-Burst Stimulation (TBS), we wanted to investigate careless responding using a real dataset. In the aforementioned study, participants were screened using the RRS (Treynor and Gonzalez, 2003) in order to assess participants’ habitual ruminative tendencies and assign them to two stratified groups (low vs. high trait ruminators).

The aims of the current investigation were to evaluate the psychometric properties of the Ruminative Response Scale, to explore the predictive value of different indicators of careless responses, and finally to evaluate the efficacy of the steps we took in changing the recruitment procedure to minimize careless responding in the first place.

## Methods

### Participants

**Figure 1:**
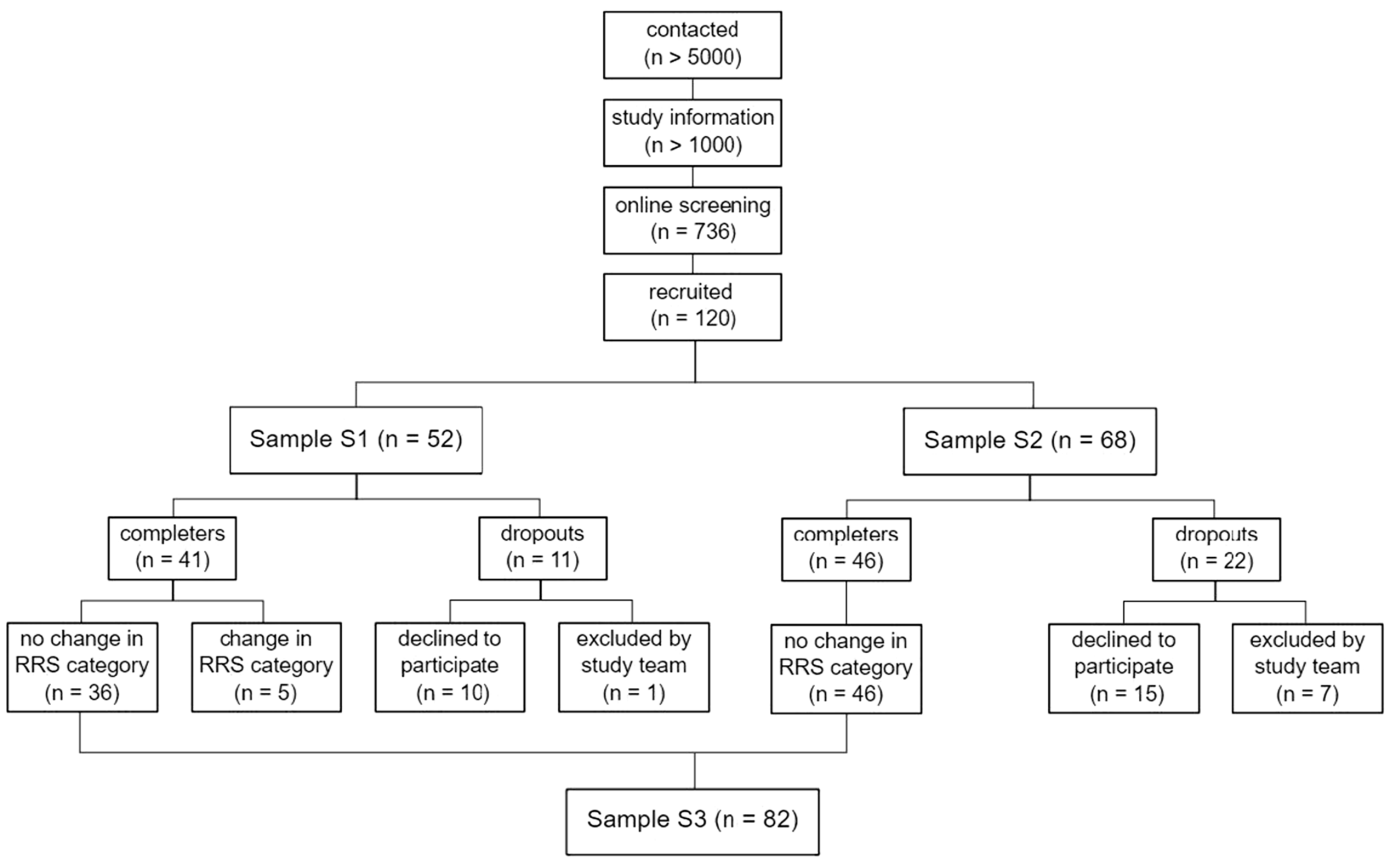
CONSORT diagram of the recruitment procedure. S1 = sample recruited prior to changing the recruitment procedure. S2 = sample recruited after changing the recruitment procedure.

The sample analyzed here was originally recruited within a larger project investigating the effects of Theta-Burst Stimulation on the stress response in low and high ruminators. For this, a total of 120 right-handed healthy volunteers were recruited via posts spread across the university hospital and social media platforms. Potentially interested participants knew there would be a stress induction and neurostimulation at each of the two appointments at the laboratory, scheduled approximately 5 weeks apart. Inclusion criteria encompassed individuals aged 18–50 with normal or corrected vision, right-handedness, absence of metal in the skull/brain, and proficiency in the German language. Exclusion criteria involved any medical conditions, including diabetes mellitus, renal insufficiency, uncontrolled hypertension, history of traumatic brain injury, cardiac arrhythmias, acute substance abuse, adrenal insufficiency, any acute psychiatric or neurological disorder, and pregnancy in women (for a list of inclusion and exclusion criteria, see Supplementary material S1). All procedures were approved by the ethics committee at the University Hospital and University of Tübingen and were in line with the Declaration of Helsinki in its latest version. Initially, an eligibility screening using an online questionnaire ( $T_{1}$ ) using SoSci Survey was completed, where demographic and clinical variables as well as the revised Ruminative Response Scale (RRS) (Treynor and Gonzalez, 2003) were assessed. According to an *a priori* power analysis for the main research question of the larger study aiming to investigate the effect of Theta-Burst Stimulation on stress-reactive rumination, we aimed to recruit a stratified sample of 44 low- and 44 high trait ruminators [low trait ruminators: mean RRS ≤ 1.82 (percentile rank 27); high trait ruminators: mean RRS ≥ 2.36 (percentile rank 65)]. For a sensitivity analysis of the reported analyses, we refer to Supplementary material S2. These corresponding cutoffs are based on the combined data of 983 participants from prior studies of our group (reference deleted for blind peer review) (Rosenbaum et al., 2018a,b, 2021). All eligible volunteers received an invitation to participate in the study. At the beginning of the first of two experimental sessions, participants completed the RRS again in paper–pencil format ( $T_{\mathrm{lab}}$ ). After completion of both appointments at the laboratory, participants were compensated with 100 € or course credit. We observed substantial changes in RRS scores from the online screening ( $T_{1}$ ) to the assessment of the RRS at the laboratory ( $T_{\mathrm{lab}}$ ) after having recruited and partly assessed a total of *n* = 52 participants. As a consequence, we changed the recruitment procedure as follows: To minimize the likelihood of the questionnaire assessing momentary states, we introduced an additional instruction before the initial online Ruminative Response Scale (RRS) assessment ( $T_{1}$ ). This instruction explicitly asked participants to consider how they typically handle negative emotions, not just how they dealt with them in the past week. We decided to do so as per this instruction because we explicitly wanted the participants to fill out the following questionnaire, especially conscientiously, to prevent misunderstandings of the questionnaire instruction as well as careless responses in general. We retained the standard RRS completion instructions both before and after adding this new instruction (see Supplementary material S3). Furthermore, we conducted telephone interviews with all participants before their inclusion in the study. During these interviews, we asked participants about their subjective opinions regarding whether they considered themselves to be low- or high trait ruminators and how they personally defined rumination. This was done to assess the alignment between their self-perceived traits and their RRS scores obtained during the initial online screening ( $T_{1}$ ). Finally, approximately 1 week before their scheduled laboratory appointments ( $T_{\mathrm{lab}}$ ), we administered the RRS as an online questionnaire once again ( $T_{2}$ ). This was done to check for significant changes between the second assessment ( $T_{2}$ ) and the initial online screening ( $T_{1}$ ). If participants were now categorized into the opposite trait group (e.g., low trait ruminators becoming high trait ruminators) or if their second RRS assessment placed them closer to the opposite group’s score range (e.g., low ruminators no longer scoring below the low RRS cutoff and approaching the high RRS cutoff), they were excluded from the study before their first laboratory appointment. These same criteria were applied when assessing the RRS at the laboratory ( $T_{\mathrm{lab}}$ ). Applying these rules to the already assessed participants, a total of seven participants substantially changed RRS scores between $T_{1}$ and $T_{\mathrm{lab}}$ . We wrote an email to all of the aforementioned participants in order to get feedback and assess the RRS again (*post-hoc* RRS) to check for the “real” group. Four out of the seven participants reported having answered the RRS at the lab regarding the current state. According to this *post-hoc* RRS, these participants were categorized as high trait ruminators, which was in line with their first assessment at $T_{1}$ . Two of the participants being asked about the category change, however, subsequently withdrew from the study. One participant was further excluded prior to the first appointment at the lab as she already filled in $T_{2}$ and changed from high to medium but closer to low. Additionally, seven participants declined to participate prior to their first appointment due to loss of interest, and one declined to participate after the first lab appointment as participation was too stressful, which resulted in 11 dropouts and *n* = 41 completers for sample S1 (see Figure 1).

After changing the recruitment procedure, another 68 participants were recruited (sample S2). The aforementioned rules resulted in five participants being excluded after $T_{2}$ , and two participants being excluded after $T_{\mathrm{lab}}$ . Out of the five, one participant was accidentally excluded by the study team after *T*_2_ due to a false calculation of the sum score, whereas there was no substantial change in RRS scores. Furthermore, a total of nine participants declined to participate prior to their first appointment at the lab due to a loss of interest in participation; four declined to participate due to the experimental session (stress induction or TBS); and two were excluded due to circulatory problems during the session. This resulted in a total of 22 dropouts and 46 completers. Considering only participants never changing categories, the final total sample (sample S3) resulted in *n* = 82 participants. For an overview of the recruitment procedure, different samples, and exclusion of participants, please find the CONSORT diagram (Moher et al., 2009) in Figure 1. For a more detailed description and illustration of changes in RRS scores, see the Results section, “Number of participants changing categories”.

#### Ruminative Response Scale

In order to assess inter-individual levels of trait rumination, the self-report Ruminative Response Scale (RRS) (Nolen-Hoeksema and Morrow, 1991), a subscale of the Response Style Questionnaire (RSQ) (Nolen-Hoeksema and Morrow, 1991), was used. The RRS consists of a total of 22 items, which are rated on 4-point Likert scales ranging from 1 = “almost never” to 4 = “almost always” and resulting in a total score ranging between 22 and 88 and consequently a mean ranging between 1 and 4. A high internal consistency has been observed in several studies and samples (Cronbach’s α > 0.88) (Nolen-Hoeksema and Morrow, 1991; Just and Alloy, 1997; Kasch et al., 2001; Moberly and Watkins, 2008), including studies using the German version of the RRS (Cronbach’s α = 0.89–0.92) (Wahl et al., 2011). Test–retest reliability, however, has been proven to fluctuate across different time spans as well as clinical and non-clinical samples: in case of non-clinical samples, test–retest reliability typically ranges between *r* = 0.80 over 6 months (Nolen-Hoeksema et al., 1994) and *r* = 0.67 over 1 year (Nolen-Hoeksema et al., 1999). In clinical samples, test–retest scores ranged between *r* = 0.36 over 6 months (Kasch et al., 2001) and *r* = 0.47 over 1 year (Just and Alloy, 1997). The original version of the Ruminative Response Scale (RRS) by Nolen-Hoeksema and Morrow (1991) was initially developed to assess depressive rumination. Treynor and Gonzalez (2003) revised the original scale due to high confounding with depression symptoms. Therefore, we used the revised scale. The psychometric properties were found to be comparable to the original RRS (Schoofs et al., 2010; Hasegawa, 2013).

#### State rumination questionnaire

We assessed stress-reactive rumination using adapted items from the RRS (Nolen-Hoeksema and Morrow, 1991), the Amsterdam Resting-State Questionnaire (Diaz et al., 2013), and the Perseverative Thinking Questionnaire (Ehring et al., 2011), as well as a questionnaire by de Jong-Meyer et al. (2009). The 18 items were answered using a 5-point Likert scale ranging from 1 (“not at all”) to 5 (“very often”), totaling a score between 18 and 90. Subjects were instructed to rate the items regarding the last 10 min. This questionnaire was used in our group in several studies (Rosenbaum et al., 2021; Laicher et al., 2023), and internal consistency has proven to be high (Cronbach’s α > 0.94) (for a full list of items, see Supplementary material S4).

#### Data analysis

In order to assess the effectiveness of our changes in the recruitment procedure, the following analyses are based on three samples. Sample S1: This sample comprises all participants who were recruited prior to the changes, excluding dropouts (*n* = 41), as well as participants who were excluded from the total sample later on due to changing RRS categories. Sample S2: Analogously, this sample comprised all participants who were recruited after changing the recruitment procedure without participants declining to participate (*n* = 53). We included all data entries of all participants until they were excluded, as in the case of the seven participants excluded by the study team. Sample S3: This sample comprised all completers that were not excluded as they never changed RRS categories.

We first report demographic variables as well as descriptive information on the mean number of days between RRS assessments. Next, we report their psychometric properties for each sample. To assess reliability, we report Cronbach’s ɑ as an index of internal consistency and the correlation of scale scores and intraclass correlations (ICC) as an index of test–retest reliability. We interpret the ICC concerning potential measurement bias (Liljequist et al., 2019) using the cutoffs proposed by Koo and Li (2016).

Finally, we fitted logistic regression models (RRS category change yes vs. no) in order to predict changes in RRS scores dependent on the defined cutoffs as described earlier. For this, we used the data of all participants assessed in the study with (*n* = 12) and without category changes (*n* = 82), while we abstained from the analysis of each sample as changing categories was a rather uncommon phenomenon and would result in fairly unequal samples. All analyses were also repeated using the R package JTRCI (Kruijt, 2023) in order to calculate Jacobson–Truax and reliable change indices and category changers identified using reliable change in contrast to the aforementioned cutoffs. However, this analysis did not yield systematically different results, which is why we included it in Supplementary material S5.

For the logistic regression models, we investigated different parameters. The report on the results is structured accordingly. First, we investigate the effect of time by fitting a model using the number of days between $T_{1}$ and $T_{\mathrm{lab}}$ as a predictor. Secondly, we investigated the predictive value of an index implemented in SPSS, the Anomaly Case Index List, which reflects the unusualness of a record with respect to the group deviation it belongs to, which is determined using cluster analysis. In a second step, we again descriptively analyzed which participants changing categories were detected according to the use of the cutoff score that was suggested by the authors of this algorithm (remove cases with an index >2). Thirdly, we investigated the predictive value of indexes of careless responses described in Curran (2016), namely the longest string of identical consecutive responses, the Intra-individual Response Variability (the standard deviation of responses across a set of consecutive item responses for an individual, i.e., between the items of the RRS), as well as the Mahalanobis distance of each participant’s RRS ratings at the respective assessment ( $T_{1}$ vs. $T_{2}$ vs. $T_{\mathrm{lab}}$ ). All of the aforementioned indexes (Anomaly Case Index, longest string, Intra-individual Response Variability, and Mahalanobis distances) are calculated by entering raw data of all RRS items per participant and, as a consequence, identifying anomalies on item level rather than total score level. Again, as a second step, we descriptively analyzed which category changers were detected as multivariate outliers by comparing the Mahalanobis distance to the critical quantile of the corresponding $\chi^{2}$ distribution. Due to the absence of meaningful cutoff scores, we abstained from such analyses in the case of the longest string and Intra-individual Response Variability. Fourthly, we analyzed response time indexes that are automatically implemented in the online tool (SoSci Survey) we were using for the assessment of $T_{1}$ and $T_{2}$ . This was, on the one hand, an index penalizing extremely fast completion (DEG_TIME), which is normed such that values of more than 100 points indicate low-quality data. On the other hand, the relative speed index, as described in Leiner (2019), is computed. After fitting the logistic regression model, we descriptively investigated the identification of category changers dependent on the cutoff scores proposed by the authors (time index ≥50 and relative speed index >2). The two time indexes are calculated using the time it took the subjects to complete the respective online questionnaire.

Fifthly, we investigated whether participants changed categories because they answered the RRS with respect to their momentary rumination, which is why we used baseline state rumination scores at $T_{\mathrm{lab}}$ and the interaction with RRS means at $T_{1}$ vs. $T_{2}$ vs. $T_{\mathrm{lab}}$ as a predictor in our logistic regression model.

Finally, in order to evaluate the effectiveness of the changes we made in the recruitment procedure, we used $\chi^{2}$ test of homogeneity (RRS category change between $T_{1}$ and $T_{\mathrm{lab}}$ yes vs. no prior to vs. after changing the recruitment procedure).

Data analysis was done using SPSS and R (R Core Team, 2023) and RStudio (RStudio Team, 2023) using the packages psych (Revelle, 2023a) and psychTools (Revelle, 2023b). We further used the packages ggplot2 (Wickham, 2016), ggThemes (Arnold, 2021), ggExtra (Attali and Baker, 2022), and networkD3 (Allaire et al., 2017) for plotting.

## Results

### Demographic data and descriptive information

**Figure 2:**
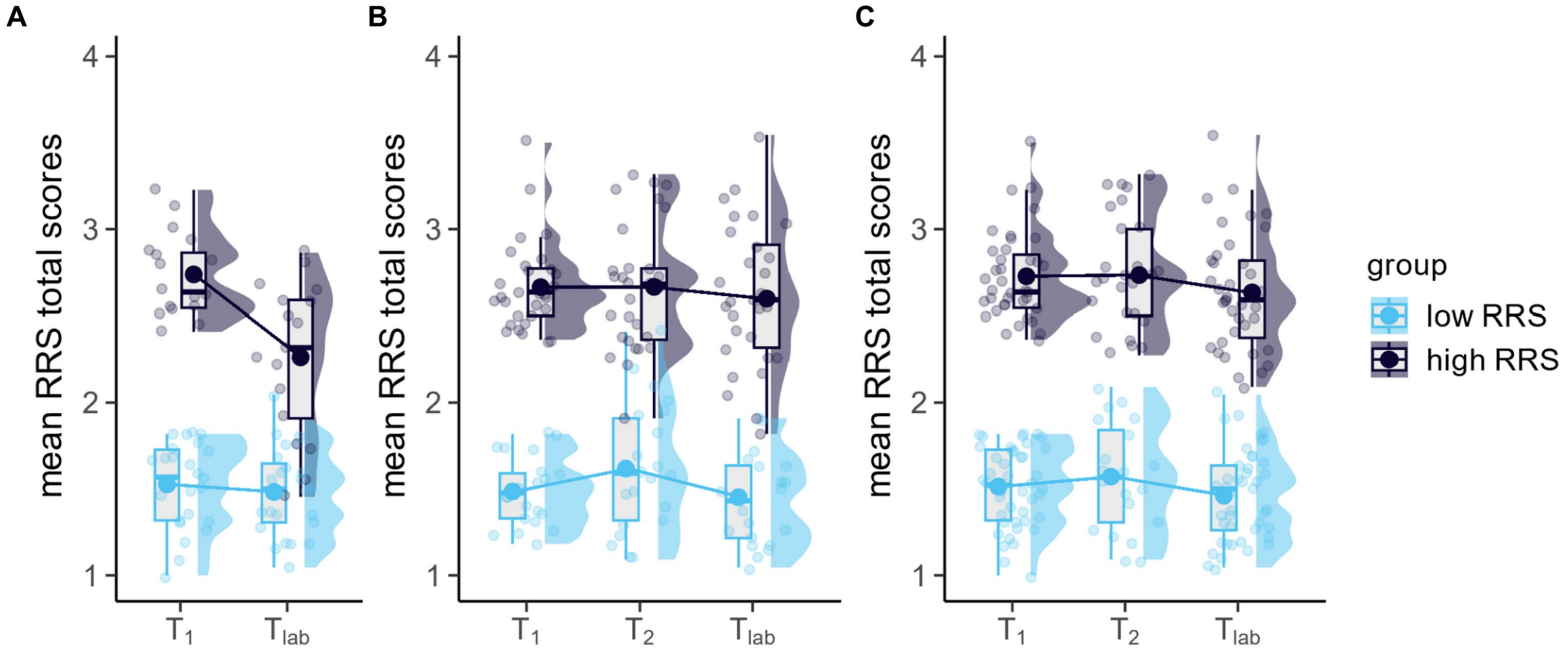
Line, boxplots, and marginal distributions of mean RRS total scores dependent on sample **(A)** Sample S1, prior to changing the recruitment procedure, **(B)** Sample S2, after changing the recruitment procedure, **(C)** Sample S3, total sample of completers never changing categories). Transparent dots indicate the raw data. Bold dots indicate the estimated marginal mean of the fitted model (time (*T_1_* vs. *T_2_* vs. *T_lab_*) × group (low RRS vs. high RRS). The lower and upper hinges of the boxplot correspond to the first and third quartiles, respectively. Whiskers extend from the hinge to the largest value, no further than 1.5 × interquartile range.

The sample prior to changing the recruitment procedure (S1, *n* = 41) comprised 75.61% females; the sample after changing the recruitment procedure (S2, *n* = 53) comprised 84.91% females; and the final total sample (S3, *n* = 82) 78.05%. The average age of the samples was *M* = 24.93 years (SD = 5.38) for S1, *M* = 23.60 years (SD = 4.31) for S2, and *M* = 24.40 years (SD = 4.94) for S3. Overall, approximately 8.5 weeks (*M* = 59.70 days, SD = 31.48 days) after the online screening ( $T_{1}$ ) was completed, participants had their appointment at the lab ( $T_{\mathrm{lab}}$ ). About 74.62 days (SD = 27.54) passed between the first ( $T_{1}$ ) and the second online assessment of the RRS ( $T_{2}$ ) and on average 5.68 days (SD = 3.62) between the second online assessment of the RRS ( $T_{2}$ ) and the appointment at the lab ( $T_{\mathrm{lab}}$ ). Samples prior to and after changes in the recruitment were comparable concerning the sex distribution, $\chi^{2}$ (1) = 0.348, *p* = 0.555, as well as their age, *F*(1, 86) = 1.660, *p* = 0.201, $\eta_{p}^{2}$ = 0.019. However, they differed concerning the average number of days between $T_{1}$ and $T_{\mathrm{lab}}$ , *F*(1, 86) = 69.850, *p* < 0.001, $\eta_{p}^{2}$ = 0.448, which was reflected by an average 37.27 days (SD = 15.43) between $T_{1}$ and $T_{\mathrm{lab}}$ in the case of the sample prior to changing the recruitment procedure (S1) compared to M = 79.28 days (SD = 28.76) between $T_{1}$ and $T_{\mathrm{lab}}$ in case of the sample recruited after the changes (S2). Concerning RRS scores, both samples were comparable concerning the distribution of mean RRS scores at $T_{1}$ , *F*(1, 86) = 1.298, *p* = 0.258, $\eta_{p}^{2}$ = 0.015, however, not concerning RRS scores at $T_{\mathrm{lab}}$ , *F*(1, 86) = 6.481, *p* < 0.05, $\eta_{p}^{2}$ = 0.070. This was reflected by overall higher RRS scores in the case of the sample after changing the recruitment procedure (*M* = 2.14, SD = 0.70) compared to prior (*M* = 1.81, SD = 0.52) (for an illustration of mean RRS scores dependent on group (low vs. high RRS) and sample (S1, S2, and S3), please see Figure 2). Further detailed information on demographic data is to be found in Table 1.

**Table 1:** Means and standard deviations of demographic variables and a summary of reliability measures dependent on each sample.

	S1 (*n* = 41)	S2 (*n* = 53)	S3 (*n* = 82)
Percentage female	75.61%	84.91%	78.05%
Age	24.93 (5.38) years	23.60 (4.31) years	24.40 (4.94) years
Number of days between $T_{1}$ and $T_{\mathrm{lab}}$	37.27 (15.43) days	79.33 (28.21) days	60.46 (30.30) days
Number of days between $T_{1}$ and $T_{2}$	–	76.80 (26.28) days	73.17 (25.99) days
Number of days between $T_{2}$ and $T_{\mathrm{lab}}$	–	5.83 (3.68) days	5.76 (3.62) days
Cronbach’s ɑ $T_{1}$	0.94 [0.91; 0.96]	0.93 [0.90; 0.96]	0.94 [0.92; 0.96]
Cronbach’s ɑ $T_{2}$	–	0.95 [0.92; 0.97]	0.95 [0.93; 0.97]
Cronbach’s ɑ $T_{\mathrm{lab}}$	0.92 [0.87; 0.95]	0.95 [0.93; 0.97]	0.95 [0.93; 0.96]
Test–retest reliability $T_{1}$ $T_{2}$	–	$r_{tt}$ = 0.84 $r_{\mathrm{ttBP}}$ = 0.67 $r_{\mathrm{ttWP}}$ = 0.59	$r_{tt}$ = 0.90 $r_{\mathrm{ttBP}}$ = 0.71 $r_{\mathrm{ttWP}}$ = 0.58
Test–retest reliability $T_{1}$ $T_{\mathrm{lab}}$	$r_{tt}$ = 0.83 $r_{\mathrm{ttBP}}$ = 0.60 $r_{\mathrm{ttWP}}$ = 0.54	$r_{tt}$ = 0.88 $r_{\mathrm{ttBP}}$ = 0.68 $r_{\mathrm{ttWP}}$ = 0.55	$r_{tt}$ = 0.90 $r_{\mathrm{ttBP}}$ = 0.69 $r_{\mathrm{ttWP}}$ = 0.55
Test–retest reliability $T_{2}$ $T_{\mathrm{lab}}$	–	$r_{tt}$ = 0.95 $r_{\mathrm{ttBP}}$ = 0.77 $r_{\mathrm{ttWP}}$ = 0.60	$r_{tt}$ = 0.96 $r_{\mathrm{ttBP}}$ = 0.78 $r_{\mathrm{ttWP}}$ = 0.61
ICC1	0.75 [0.57; 0.86]	0.88 [0.81; 0.92]	0.91 [0.88; 0.94]
ICC2	0.75 [0.47; 0.88]	0.88 [0.81; 0.92]	0.91 [0.87; 0.94]
ICC3	0.80 [0.66; 0.89]	0.88 [0.82; 0.92]	0.92 [0.88; 0.94]

Sample S1: prior to changing the recruitment procedure; Sample S2: after changing the recruitment procedure; Sample S3: total sample of completers never changing categories. A full summary of different ICCs for each sample is to be found in Supplementary material S6. $r_{tt}$ = overall test–retest reliability (correlation of scale scores over time), $r_{\mathrm{ttBP}}$ = mean between person, across item reliability, $r_{\mathrm{ttWP}}$ = mean within person, across item reliability.

### Number of participants changing categories

In the case of sample S1 (*n* = 41), 23 participants (56.1% of the sample) were categorized as low trait ruminator at $T_{1}$ and remained low at $T_{\mathrm{lab}}$ while eight high trait ruminators (19.5% of the sample) remained high at $T_{\mathrm{lab}}$ . Four participants (9.8%) changed from high at $T_{1}$ to low at $T_{\mathrm{lab}}$ , another four participants (9.8%) changed from high at $T_{1}$ to medium but remained closer to high at $T_{\mathrm{lab}}$ while one participant (2.4%) changed from high to medium but closer to low at $T_{\mathrm{lab}}$ . Finally, one participant (2.4%) originally categorized as a low trait ruminator changed to medium but remained closer to low. Accordingly, following the rules of recruitment applied in the case of sample S2, five (12.2%) participants changed categories from $T_{1}$ to $T_{\mathrm{lab}}$ .

**Figure 3:**
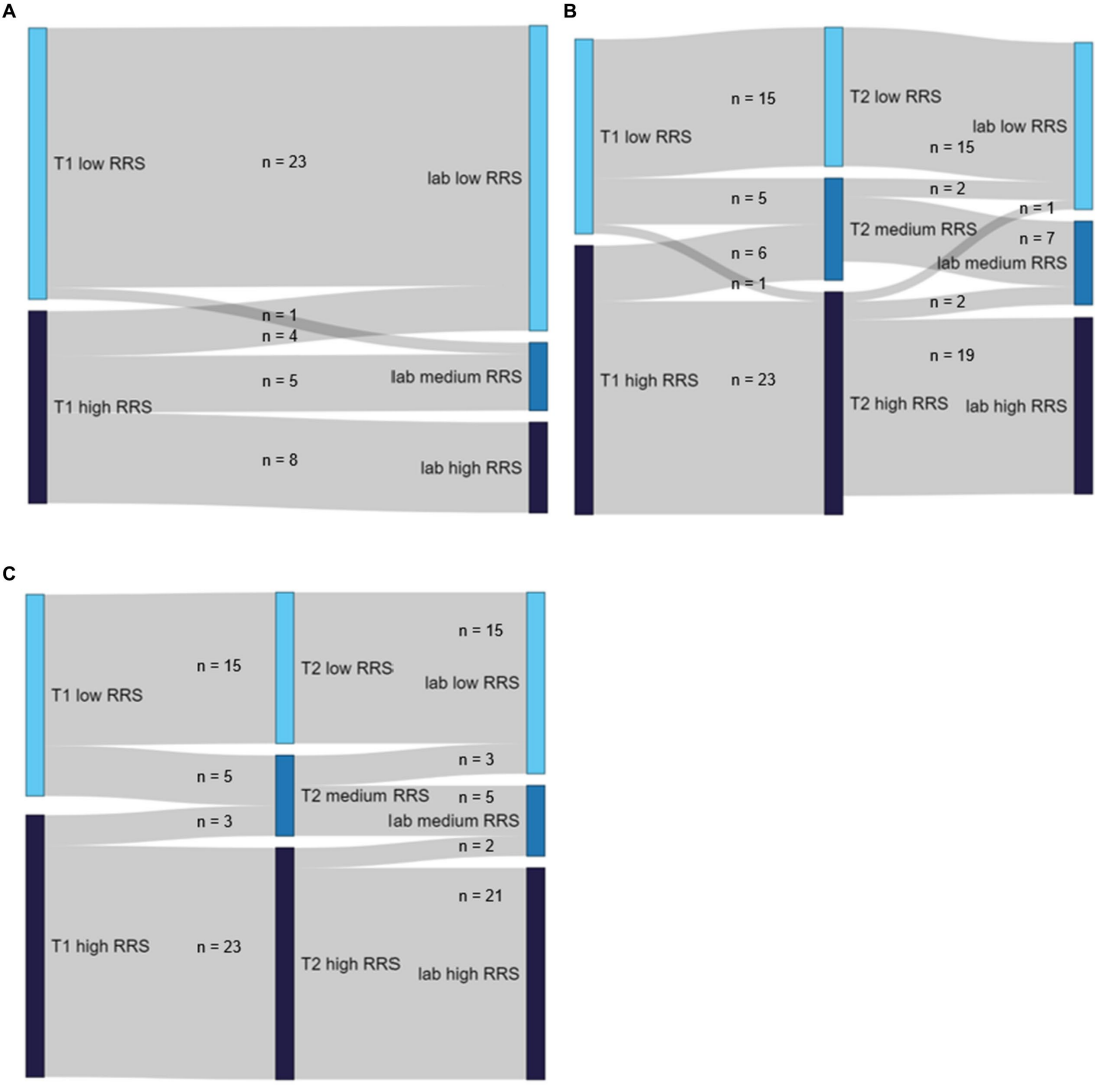
Sankey diagrams illustrating the changes of mean RRS scores and corresponding groups dependent on sample **(A)** Sample S1. **(B)** Sample S2. **(C)** Sample S3.

In the case of sample S2 (*n* = 53), 21 participants (39.6%) who were high trait ruminators at $T_{1}$ remained in this group also at $T_{2}$ and $T_{\mathrm{lab}}$ . Analogously, this was the case for 15 (28.3%) low trait ruminators at $T_{1}$ which remained in this group also at $T_{2}$ and $T_{\mathrm{lab}}$ . Five (9.4%) participants that were formerly grouped as low trait ruminators at $T_{1}$ fell between the cutoffs but closer to their original group at $T_{2}$ . Three out of those five changed to the low group again at $T_{\mathrm{lab}}$ (5.7%), two remained medium but closer to low (3.8%). Two high trait ruminators (3.8%) remained high at $T_{2}$ but eventually fell between the cutoffs but closer to high at $T_{\mathrm{lab}}$ . Three participants (5.7%) who were formerly grouped as high trait ruminators at $T_{1}$ fell between the cutoffs but closer to their original group at $T_{2}$ and remained medium but closer to high. Two (3.8%) further high trait ruminators at $T_{1}$ fell between the cutoffs but closer to their original group at $T_{2}$ but were excluded at $T_{\mathrm{lab}}$ after falling between the cutoffs but closer to low. One participant (1.9%) was excluded at $T_{\mathrm{lab}}$ after changing to the low trait ruminator group albeit being recruited as and remaining a high trait ruminator at $T_{2}$ . Four (7.5%) participants were excluded after $T_{2}$ : one participant after changing from low to high, one participant after changing from low to medium but closer to high, one participant after changing from high to medium but closer to low, and one participant after changing from high to low. One high trait ruminator was excluded after $T_{2}$ due to a false calculation of the sum score whereas there was no substantial change in RRS scores. For an illustration of the changes of RRS means and corresponding groups, see Figure 3.

In the case of sample S3 (*n* = 82), where all participants changing categories were excluded, a total of 38 participants (46.3%) remained low trait ruminators throughout all RRS assessments (23 assessed only two times at $T_{1}$ and $T_{\mathrm{lab}}$ , 15 three times at $T_{1}$ , $T_{2}\text{,}$ and $T_{\mathrm{lab}}$ ), while this was true for 29 high trait ruminators (35.4%) (eight assessed two times at $T_{1}$ and $T_{\mathrm{lab}}$ , 21 three times at $T_{1}$ , $T_{2}$ and $T_{\mathrm{lab}}$ ). A total of six participants (7.3%) being categorized as low trait ruminators at $T_{1}$ changed to medium trait ruminators (but closer to the low compared to the high cutoff) at the next RRS assessment (one assessed two times at $T_{1}$ and $T_{\mathrm{lab}}$ , five assessed three times at $T_{1}$ , $T_{2}$ and $T_{\mathrm{lab}}$ ). Out of the five being assessed three times, three changed again to the low group, while two remained medium ruminators at $T_{\mathrm{lab}}$ . Analogously, seven high trait ruminators at $T_{1}$ (8.5%) changed from high to medium at the next RRS assessment (four assessed two times at $T_{1}$ and $T_{\mathrm{lab}}$ , three assessed three times at $T_{1}$ , $T_{2}$ , and $T_{\mathrm{lab}}$ ). All of the three participants being assessed three times remained medium trait ruminators at $T_{\mathrm{lab}}$ . Finally, two participants (2.5%) remained high trait ruminators at $T_{1}$ and $T_{2}$ but fell in between the cutoffs at $T_{\mathrm{lab}}$ .

#### Predicting change in RRS categories

##### Time

Fitting our first model using only the number of days between $T_{1}$ and $T_{\mathrm{lab}}$ as a predictor, we found that time between measurements did not predict changing RRS categories ( β = −0.004, z = −0.289, *p* = 0.772).

##### SPSS Anomaly Index

Next, we fitted three models, each including the Anomaly Case Index generated by SPSS for the respective RRS assessment ( $T_{1}$ vs. $T_{2}$ vs. $T_{\mathrm{lab}}$ ). While the Anomaly Index of $T_{1}$ ( β = −0.136, z = −0.103, *p* = 0.918) and the Anomaly Index of $T_{2}$ did not yield significant predictors ( β = 1.883, z = 1.293, *p* = 0.196), the Anomaly Index of $T_{\mathrm{lab}}$ ( β = 2.422, z = 1.953, *p* = 0.051) yielded marginal significance. That means, descriptively, for a one-unit increase in the Anomaly Index of $T_{\mathrm{lab}}$ , the odds of changing RRS categories (vs. not) increase by a factor of 11.26. According to the authors of the algorithm, observations with Anomaly Case Indexes >2 should be excluded. Descriptively, this was only the case for the RRS score at $T_{2}$ of one participant (out of a total of eight participants changing categories between $T_{1}$ and $T_{\mathrm{lab}}$ ) who was excluded at the lab after changing categories between $T_{1}$ (high), $T_{2}$ (medium but closer to high) and $T_{\mathrm{lab}}$ (medium but closer to low). For crosstables of the absolute and relative frequency participants changing and not changing categories were flagged, see Table 2.

**Table 2:** Frequency of category changers being flagged by SPSS Anomaly Indices.

			Category change		
Index	Identification rate		No	Yes	Total
SPSS Anomaly Index $T_{1}$	Not flagged	Count	92	14	106
		% within SPSS Anomaly Index $T_{1}$	86.8%	13.2%	100.0%
		% within category change	100.0%	100.0%	100.0%
		% of total	86.8%	13.2%	100.0%
	Flagged	Count	0	0	0
		% within SPSS Anomaly Index $T_{1}$	0.0%	0.0%	0.0%
		% within category change	0.0%	0.0%	0.0%
		% of total	0.0%	0.0%	0.0%
	Total	Count	92	14	106
		% within SPSS Anomaly Index $T_{1}$	86.8%	13.2%	100.0%
		% within category change	100.0%	100.0%	100.0%
		% of total	86.8%	13.2%	100.0%
SPSS Anomaly Index $T_{2}$	Not flagged	Count	55	6	61
		% within SPSS Anomaly Index $T_{2}$	90.2%	9.8%	100.0%
		% within category change	100.0%	85.7%	98.4%
		% of total	88.7%	9.7%	98.4%
	Flagged	Count	0	1	1
		% within SPSS Anomaly Index $T_{2}$	0.0%	100.0%	100.0%
		% within category change	0.0%	14.3%	1.6%
		% of total	0.0%	1.6%	1.6%
	Total	Count	55	7	62
		% within SPSS Anomaly Index $T_{2}$	88.7%	11.3%	100.0%
		% within category change	100.0%	100.0%	100.0%
		% of total	88.7%	11.3%	100.0%
SPSS Anomaly Index $T_{\mathrm{lab}}$	Not flagged	Count	86	10	96
		% within SPSS Anomaly Index $T_{\mathrm{lab}}$	89.6%	10.4%	100.0%
		% within category change	100.0%	100.0%	100.0%
		% of total	89.6%	10.4%	100.0%
	Flagged	Count	0	0	0
		% within SPSS Anomaly Index $T_{\mathrm{lab}}$	0.0%	0.0%	0.0%
		% within category change	0.0%	0.0%	0.0%
		% of total	0.0%	0.0%	0.0%
	Total	Count	86	10	96
		% within SPSS Anomaly Index $T_{\mathrm{lab}}$	89.6%	10.4%	100.0%
		% within category change	100.0%	100. 0.0%	100.0%
		% of total	89.6%	10.4%	100.0%

##### Careless package

Next, we used indexes of the careless package implemented in R. Entering the corresponding indices of the respective RRS assessments ( $T_{1}$ vs. $T_{2}$ vs. $T_{\mathrm{lab}}$ ) separately and as an interaction with each other did not yield any significant predictor. When fitting logistic regression models using the Mahalanobis distances of the respective RRS assessment ( $T_{1}$ vs. $T_{2}$ vs. $T_{\mathrm{lab}}$ ), we did not observe any significant predictors (all p’s > 0.326). Next, we investigated the Mahalanobis distances descriptively: Comparing RRS ratings at $T_{1}$ with the critical quantile for ɑ = 0.05, 14 out of 95 participants were identified as multivariate outliers. Out of these 14, two participants out of a total of 12 category changers were identified [one changing between $T_{1}$ and $T_{2}$ (out of a total of four changing; 25% identified); one changing between $T_{1}$ and $T_{\mathrm{lab}}$ (out of a total of eight participants changing; 12.5% identified)]. Concerning, RRS ratings at $T_{2}$ 4 out of 54 participants were identified as multivariate outliers. Out of these four, two participants (out of a total of four participants changing categories between $T_{1}$ and $T_{2}$ ) were identified as multivariate outliers (*p* < 0.05). One of the two participants has already been identified as a multivariate outlier according to the Mahalanobis distance of RRS ratings at $T_{1}$ . The other one was also identified by the Anomaly Index of SPSS at $T_{2}$ . For RRS ratings at $T_{\mathrm{lab}}$ , 12 out of 95 participants were identified as multivariate outliers (*p* < 0.05). Out of those 12, two participants changing categories between $T_{1}$ and $T_{\mathrm{lab}}$ were identified whereas one of those was the participant already identified as a multivariate outlier concerning his RRS ratings at $T_{1}$ and $T_{2}$ (see Table 3).

**Table 3:** Frequency of category changers being flagged by Mahalanobis distances (MAD).

			Category change		
Index	Identification rate	MAD	No	Yes	Total
MAD $T_{1}$	Not flagged	Count	71	10	81
		% within MAD $T_{1}$	87.7%	12.3%	100.0%
		% within category change	85.5%	83.3%	85.3%
		% of total	74.7%	10.5%	85.3%
	Flagged	Count	12	2	14
		% within MAD $T_{1}$	85.7%	14.3%	100.0%
		% within category change	14.5%	16.7%	14.7%
		% of total	12.6%	2.1%	14.7%
	Total	Count	83	12	95
		% within MAD $T_{1}$	87.4%	12.6%	100.0%
		% within category change	100.0%	100.0%	100.0%
		% of total	87.4%	12.6%	100.0%
MAD $T_{2}$	Not flagged	Count	45	5	50
		% within MAD $T_{2}$	90.0%	10.0%	100.0%
		% within category change	95.7%	71.4%	92.6%
		% of total	83.3%	9.3%	92.6%
	Flagged	Count	2	2	4
		% within MAD $T_{2}$	50.0%	50.0%	100.0%
		% within category change	4.3%	28.6%	7.4%
		% of total	3.7%	3.7%	7.4%
	Total	Count	47	7	54
		% within MAD $T_{2}$	87.0%	13.0%	100.0%
		% within category change	100.0%	100.0%	100.0%
		% of total	87.0%	13.0%	100.0%
MAD $T_{\mathrm{lab}}$	Not flagged	Count	68	6	74
		% within MAD $T_{\mathrm{lab}}$	91.9%	8.1%	100.0%
		% within category change	82.9%	75.0%	82.2%
		% of total	75.6%	6.7%	82.2%
	Flagged	Count	14	2	16
		% within MAD $T_{\mathrm{lab}}$	87.5%	12.5%	100.0%
		% within category change	17.1%	25.0%	17.8%
		% of total	15.6%	2.2%	17.8%
	Total	Count	82	8	90
		% within MAD $T_{\mathrm{lab}}$	91.1%	8.9%	100.0%
		% within category change	100.0%	100.0%	100.0%
		% of total	91.1%	8.9%	100.0%

##### SoSci survey response time indexes

Finally, we fitted logistic regression models using the time indexes of the SoSci Survey (consequently, they were only available for the online assessments at $T_{1}$ and $T_{2}$ ). Again, we entered each predictor on its own and as an interaction of both predictors; however, we observed no significant effects (all p’s > 0.140). We first investigated the time index and the proposed cutoff of time index ≥50. This resulted in $T_{1}$ ratings of 15 participants out of 95 in total being flagged. Out of these 15, three participants changed categories: Two changed categories between $T_{1}$ and $T_{\mathrm{lab}}$ while in one of them $T_{2}$ ratings were not available as this participant was assessed prior to the changes in recruitment procedure. Those two participants were also identified by the Mahalanobis distances and one of them also using the SPSS Anomaly Index. In the case of $T_{2}$ ratings, 4 out of 54 participants were flagged, however, none of them changed categories at any time. When using proposed cutoff score of the relative speed index (relative speed index >2), one participant’s $T_{1}$ ratings and one participant’s $T_{2}$ ratings were flagged, however, none of them were category changers (see Tables 4, 5).

**Table 4:** Frequency of category changers being flagged by Time_Deg index.

			Category change		
Index	Identification rate		No	Yes	Total
Time_Deg $T_{1}$	Not flagged	Count	71	9	80
		% within Time_Deg $T_{1}$	88.8%	11.3%	100.0%
		% within category change	85.5%	75.0%	84.2%
		% of total	74.7%	9.5%	84.2%
	Flagged	Count	12	3	15
		% within Time_Deg $T_{1}$	80.0%	20.0%	100.0%
		% within category change	14.5%	25.0%	15.8%
		% of total	12.6%	3.2%	15.8%
	Total	Count	83	12	95
		% within Time_Deg $T_{1}$	87.4%	12.6%	100.0%
		% within category change	100.0%	100.0%	100.0%
		% of total	87.4%	12.6%	100.0%
Time_Deg $T_{2}$	Not flagged	Count	43	7	50
		% within Time_Deg $T_{2}$	86.0%	14.0%	100.0%
		% within category change	91.5%	100.0%	92.6%
		% of total	79.6%	13.0%	92.6%
	Flagged	Count	4	0	4
		% within Time_Deg $T_{2}$	100.0%	0.0%	100.0%
		% within category change	8.5%	0.0%	7.4%
		% of total	7.4%	0.0%	7.4%
	Total	Count	47	7	54
		% within Time_Deg $T_{2}$	87.0%	13.0%	100.0%
		% within category change	100.0%	100.0%	100.0%
		% of total	87.0%	13.0%	100.0%

Time_Deg = Index implemented in SoSci Survey penalizing fast response times.

**Table 5:** Frequency of category changers being flagged by RSI index.

			Category change		
Index	Identification rate		No	Yes	Total
RSI $T_{1}$	Not flagged	Count	82	12	94
		% within RSI $T_{1}$	87.2%	12.8%	100.0%
		% within category change	98.8%	100.0%	98.9%
		% of total	86.3%	12.6%	98.9%
	Flagged	Count	1	0	1
		% within RSI $T_{1}$	100.0%	0.0%	100.0%
		% within category change	1.2%	0.0%	1.1%
		% of total	1.1%	0.0%	1.1%
	Total	Count	83	12	95
		% within RSI $T_{1}$	87.4%	12.6%	100.0%
		% within category change	100.0%	100.0%	100.0%
		% of total	87.4%	12.6%	100.0%
RSI $T_{2}$	Not flagged	Count	46	7	53
		% within RSI $T_{2}$	86.8%	13.2%	100.0%
		% within category change	97.9%	100.0%	98.1%
		% of total	85.2%	13.0%	98.1%
	Flagged	Count	1	0	1
		% within RSI $T_{2}$	100.0%	0.0%	100.0%
		% within category change	2.1%	0.0%	1.9%
		% of total	1.9%	0.0%	1.9%
	Total	Count	47	7	54
		% within RSI $T_{2}$	87.0%	13.0%	100.0%
		% within category change	100.0%	100.0%	100.0%
		% of total	87.0%	13.0%	100.0%

RSI = Relative Speed Index (index implemented in SoSci Survey).

##### Baseline state rumination

In order to investigate a potential bias in RRS ratings due to a confound with current state rumination, we finally fitted logistic regression models using the interaction of RRS scores at $T_{1}$ vs. $T_{2}$ vs. $T_{\mathrm{lab}}$ with baseline state rumination ratings at $T_{\mathrm{lab}}$ . As a result, we observed marginally significant effects in the case of the main effect of RRS score at $T_{1}$ ( β = 8.282, z = 1.920, *p* = 0.055) and the interaction effect of RRS score at $T_{1}$ and state rumination ( β = −3.935, z = −1.653, *p* = 0.098). For every one-unit change in RRS scores at $T_{1}$ , the log odds of changing categories versus not increases by 8.28, and descriptively, the effect of RRS scores on the odds of changing categories versus not decreases in case of higher state rumination levels at the lab (and the other way around: the effect of state rumination at the lab decreases in case of higher RRS scores at $T_{1}$ ).

### Effectiveness of the changes made in the recruitment procedure

Finally, we investigated whether the distribution of category changers was different prior to vs. after changing the recruitment procedure, which turned out to not be the case, $\chi^{2}$ (1) = 1.254, *p* = 0.263.

## Discussion

This investigation aimed to evaluate the predictive value of different indexes for careless responses. Out of all indexes analyzed, Mahalanobis distances seem to be an easy-to-use tool with an acceptable trade-off of sensitivity and specificity that is applicable in most cases.

Scientific progress inevitably requires that findings build on each other. In particular, research in applied subjects often relies on fundamentals that have not yet been fully explored or are currently being investigated. The data presented in this article may serve as a reminder of this fact and bring more awareness to the corresponding consequences. In a recent study of our group, we aimed to investigate the neural correlates of rumination in response to social stress and the impact of Theta-Burst Stimulation over the left dorsolateral prefrontal cortex. For this, participants were screened using a trait questionnaire of ruminative thinking [Ruminative Response Scale (RRS); Treynor and Gonzalez, 2003] in order to assign them to two stratified groups (low- vs. high trait ruminators). These groups were assumed to differ regarding their habitual tendency to ruminate; however, administering the same questionnaire again at the beginning of the experimental session yielded significant changes. As trait measures are generally assumed to have a negligible amount of situation-specific variance, we expected more or less concordant RRS scores. It is further assumed that participants are comparable according to the traits indicated by the questionnaire scores. In the absence of any systematic differential item functioning, this implicates that participants having the same total scores in the RRS are comparable according to their tendency to ruminate (DeVellis, 2006). Whereas the philosophical debate concerning a person’s ability to introspectively assess their own habitual tendencies regarding their whole lifetime and the comparability of this introspection across everyone else is out of the scope of this article, these axioms should also be kept in mind as they underlie the aforementioned stratification and recruitment.

Being confronted with this non-negligible amount of variance, we aimed to investigate potential predictors of participants substantially changing in trait measures and report practical implications for other researchers. As we became aware of this problem throughout the data collection of the aforementioned study, we changed the recruitment procedure henceforth as follows: We introduced more “manipulation checks” for consistent answering in order to eliminate potential misunderstandings. For instance, by adding an extra disclaimer to the online screening stating that participants should think about how they typically handle negative emotions and not just regarding, for instance, the last 2 weeks, there is a chance of participants being more aware of this questionnaire aiming to assess a habitual tendency, a trait, rather than a momentary state. We further checked for misunderstandings of the term “rumination” using a telephone interview and introduced another RRS online assessment 1 week prior to the lab appointment. Unfortunately, we changed several aspects of the recruitment and experimental procedures at once, which is why we are unable to differentiate the efficacy and impact of each change regarding the reduction of careless responses. We did this because we attempted to induce a strong reduction in careless responses as much as possible. In the following, we report our results dependent on samples prior to and after changing the recruitment procedure, as well as the total sample.

We first analyzed the reliability of the different RRS assessments in order to investigate potential conspicuities compared to the psychometric properties reported in the literature. While Cronbach’s ɑ was nearly the same across the RRS assessments and samples, test–retest reliability seemed to substantially differ. While equal ICC values dependent on ICC type indicate the absence of systematic error, which was the case when we analyzed the final total sample where participants changing categories were excluded, there were different ICC values indicating systematic error, especially in the case of the sample assessed prior to changing the recruitment procedure.

As a next step, we investigated predictors of participants whose RRS scores substantially changed within the weeks between the different assessments by fitting logistic regression models (category change: yes vs. no). The number of days, i.e., the sheer amount of time between assessments, had no significant effect on whether participants switched categories, which is consistent with the assumption that the RRS claims to measure a trait that should remain relatively stable over time. Considering the frequency and number of iterations used in our case, this also implicates a negligible effect of memorizing previous answers.

Next, we investigated different indexes of careless responses provided by the software used to collect the data as well as implemented in the software used to analyze the data. This comprised, for instance, an index of the unusualness of cases according to a cluster analysis, namely the Anomaly Case Index generated by SPSS. Using this index and the proposed cutoff scores, we identified the RRS ratings at the second online assessment of one participant who changed RRS categories. While the sensitivity of this index was really low as a result of the number of participants and not changing RRS categories being flagged, the specificity was rather high. Two other indexes used response times to identify careless responses. Again, both did not yield significant predictors in our fitted logistic regression models; however, using the proposed cutoff scores, $T_{1}$ ratings of 15 participants out of 95 (~16%) in total were flagged. A third of those indeed changed categories later in the study.

As a last step, we used several indices proposed by Curran (2016) and implemented in the statistical software R (R Core Team, 2023), namely the longest string of identical consecutive responses, the Intra-individual Response Variability, and the Mahalanobis distance of each participant’s RRS ratings at the respective assessments. While none of these indexes yielded significant predictors of category change, Mahalanobis distances were able to identify several participants changing categories. Albeit rather many participants were flagged in general (~14% of all participants’ RRS ratings at $T_{1}$ , ~7% of all participants RRS ratings at $T_{2}$ and ~ 12% of all participants RRS ratings at $T_{\mathrm{lab}}$ ), up to 50% of all participants changing categories after the respective RRS assessment were identified. Especially in the light of the aforementioned results of the other indexes proposed in the literature, Mahalanobis distances seem to be an easy-to-use tool with an acceptable trade-off of sensitivity and specificity that is applicable in the case of paper–pencil as well as online questionnaire data and further does not require the use of a commercial software.

As several participants changing categories qualitatively reported to have answered the RRS as a state measure, we further conducted an analysis investigating the predictive value of baseline state rumination, which was assessed shortly after participants completed the RRS at the laboratory session. We observed a marginally significant main effect of RRS score at $T_{1}$ and interaction of RRS score at $T_{1}$ and state rumination, which suggests that there is most probably not only one underlying cause of the observed changes in RRS scores. Presumably, some participants reliably answer the RRS as a state measure, while others show careless responses to some degree, and others might show distortions due to other reasons. As we observed a significant impact of state rumination, future studies should further assess state rumination concurrently with trait rumination in order to disentangle these interrelationships. The precise reasons for these substantially differing RRS scores remain unclear. And while we investigated some potential predictors in this analysis, our sample size is too small to give an answer to the question of the source of the variability. What these analyses, however, are able to show is the need for predetermined rules, a “manipulation check,” in order to assess variation in the data regardless of the cause. Like this, corresponding participants can be excluded prior to their lab appointment, and results are more trustworthy compared to data where underlying assumptions have never been tested. Ultimately, we cannot assume participants not changing categories to be “better” or “more reliable” only because there is less intra-person variability. Most probably, additional qualitative data could be a valuable source of information concerning the motivation of participants (e.g., asking about thoughts during resting/waiting phases). While some participants might report thinking about the aim and method of the study, there might also be first hints to participants reporting not caring and “doing it all solely for the monetary compensation.” We asked participants about these changes in their RRS scores, and while some reported having answered the RRS as a state and their ratings being tinted by their current bad mood, others have reported that they have not “learnt the questionnaire by heart.” Specially to prevent participants from answering the RRS as a state by accident, we introduced an instruction to conscientiously read the following instructions and complete the questionnaire. In addition to potentially increased data quality, this however, might also have unintended consequences, like, for instance, participants taking longer to complete the questionnaire, which in turn has an impact on the analyzed time indexes generated by the SoSci Survey. Furthermore, participants thinking about their answers more thoroughly might make their answers incomparable to those of participants who also completed the questionnaire truthfully and in accordance with the instructions, but rather intuitively. It would be an interesting endeavor to estimate the credibility of the data assessed *post-hoc* using, for instance, the Randomized Response Technique (Lensvelt-Mulders et al., 2005); however, this is beyond the scope of this article.

One major limitation of the current investigation is the sample size. Although comparable to other studies in psychological research, the rather seldom occurrence of category-changing participants (i.e., little variance to be explained) and consequently different sample sizes (category change vs. no category change) is most probably one reason for problems in fitting our logistic regression models. The distinction of “category change” further followed a rather arbitrary cutoff and the reliable change index, while numeric changes of questionnaire scores might also be of particular interest. As this investigation, however, resulted from a stratified sampling, we found this approach the most appropriate. The assessment of two extreme groups (low and high ruminators) rather than a larger sample also including medium ruminators, however, prevents conclusions that can be drawn for the moderate group into which most of the normal population falls. Another limitation to keep in mind is that simultaneous changes were made to recruitment procedures, instructions, and other aspects, making it challenging to pinpoint the exact cause of the variations in result stability. When evaluating the efficacy of the changes made, we found no significant differences in the distribution of category changers prior to and after changing the recruitment procedure. Future research should systematically alter individual aspects to isolate their effects, explore alternative and potentially more efficient adjustments, and consider potential differential impacts resulting from the interplay of various modifications. A last point to consider is further the variance induced via the form of assessment: face-to-face and online assessments of questionnaires each come with their own set of advantages and disadvantages. Especially important in the context of the current investigation is that the former allows for clarifications and a controlled environment, while online assessments, for instance, offer metadata on completion time (Evans and Mathur, 2018). For a comprehensive discussion of the advantages and disadvantages of administration format with a focus on personality questionnaires, see Hertel et al. (2002). This study, however, was not primarily designed to systematically investigate inattentive responses but to report the usability of several indexes of careless responses using a typical clinical dataset in psychological science and give practical implications for other researchers. Further studies concerning the identification of careless responses are desperately needed. Moreover, scientists and readers should be aware of the underlying assumptions that are made using, for instance, a single “trait” questionnaire in order to stratify groups that are believed to differ concerning their responses. Recent investigations aimed at evaluating the extent of trait and state aspects in well-known questionnaires such as Spielberger’s state and trait anxiety and anger scales (Lance et al., 2021) found state and trait assessments were both dominated by stable trait-like variance. This gives rise to reopening the discussion of shared and unique variance of state and trait measures in general, as well as the need for corresponding scrutinizing of well-established measures. New tools, such as ecological momentary assessments of a variable of interest, might be used to screen participants prior to study inclusion for a more reliable differentiation of traits and states. One promising approach might also be latent state–trait theory (Steyer et al., 1992; Geiser et al., 2017), which has been proven to be superior to classical test theory in individual change detection (Jabrayilov et al., 2016).

Summing it up, the authors would like to raise awareness about the topic of careless responses, which many researchers are not thoroughly aware of when it comes to their own data. We would like to emphasize the importance of manipulation checks using, for instance, multiple assessments of questionnaires and different data sources (e.g., qualitative data, interviews prior to study inclusion). While it may not be feasible for all studies to implement manipulation checks in the first place, *post-hoc* investigation of potential careless responding is always applicable (DeSimone and Harms, 2018). Following our results, we recommend conducting Mahalanobis distance analyses to identify multivariate outliers, allowing researchers to assess the credibility of their results as the comparability of studies with different and undetected relative frequencies of reliable data is most probably limited and might also be one cause of failed replications (Maniaci and Rogge, 2014).

## Data availability statement

The raw data supporting the conclusions of this article will be made available upon request by the authors, without undue reservation.

## Ethics statement

The studies involving humans were approved by Ethics Committee at the University Hospital and University of Tübingen. The studies were conducted in accordance with the local legislation and institutional requirements. The participants provided their written informed consent to participate in this study. Written informed consent was obtained from the individual(s) for the publication of any potentially identifiable images or data included in this article.

## Author contributions

II-V: Conceptualization, Data curation, Formal analysis, Investigation, Methodology, Visualization, Writing – original draft, Writing – review & editing. A-CE: Conceptualization, Funding acquisition, Project administration, Resources, Supervision, Validation, Writing – original draft, Writing – review & editing. AF: Conceptualization, Funding acquisition, Project administration, Resources, Supervision, Validation, Writing – original draft, Writing – review & editing. DR: Conceptualization, Data curation, Formal analysis, Funding acquisition, Investigation, Methodology, Project administration, Resources, Supervision, Validation, Writing – original draft, Writing – review & editing.
