# Supplementary material for: On assessing trait rumination using the Ruminative Response Scale
Source: Front Psychol. 2024 Jun 5;15:1368390. doi: 10.3389/fpsyg.2024.1368390 (PMC11186473; doi:10.3389/fpsyg.2024.1368390)
Supplement: Supplementary file 1 [file Data_Sheet_1.pdf]

Supplementary material

Supplementary material S1:

Inclusion and exclusion criteria

Inclusion criteria:

- Age between 18 and 50 years
- Normal vision (or appropriate correction)
- Right-handedness
- No metal in the skull / brain
- German as native language or very good knowledge of German

Exclusion criteria:

- Diabetes mellitus
- renal insufficiency
- non-adjusted hypertension
- history of traumatic brain injury
- cardiac arrhythmias
- acute substance abuse
- adrenal insufficiency
- any acute psychiatric or neurological disorder (including any anomalies in the SCID-Screening (Structured Clinical Interview; First et al., 2015)
- in case of women: pregnancy

Supplementary material S2:

Sensitivity of the performed analysis (achieved power dependent on the sample size)

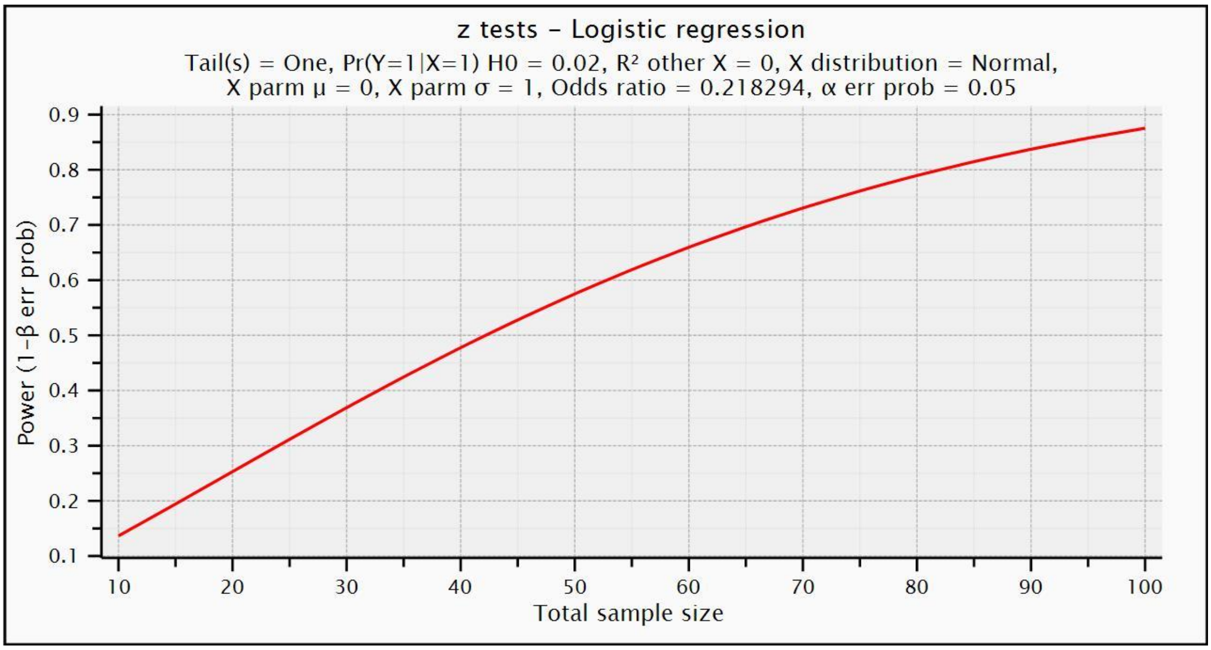

Supplementary material S3:
Instructions of the RRS

General instruction of the RRS, that was included prior as well as after changing the recruitment procedure:

“People think and behave very differently when they feel sad or down. For all of the following statements, please indicate whether you "almost never," "sometimes," "often," or "almost always" think or do them when you felt sad, down, or depressed. Please indicate what you usually do, not what you think you should do.”

Additional instruction of the RRS, that was added after changing the recruitment procedure:

“Please fill out the following questionnaire especially conscientiously: It is the last page of the screening questionnaire. Attention, the questionnaire is intended to record how you usually deal with negative emotions. Therefore, try to take your time to think about it and do not try to answer the questions solely in relation to the last week, for example, but think about your basic handling of negative emotions.”

Supplementary material S4:
Items of the state rumination questionnaire in German and their translation in English

Scoring: The items are rated on a 5-point Likert-Scale (1 = "not at all", 2 = "almost not", 3 = "a little", 4 = "often" and 5 = "very often"). Please note that items 7 and 13 are reverse-coded. The sum-score is consequently calculated as follows: item1 + item2 + item3 + item4 + item5 + item6 + (6-item7) + item8 + item9 + item10 + item11 + item12 + (6-item13) + item14 + item15 + item16 + item17 + item18

Item	German translation	English translation
	Im Folgenden werden Ihnen Fragen zur letzten Ruhephase gestellt. Wir bitten Sie anzugeben, inwiefern die folgenden Aussagen über Ihr Erleben während dieser Zeit übereinstimmen. Sie können hierzu die folgenden Einschätzungen abgeben: „gar nicht“, „fast nicht“, „ein wenig“, „oft“ und „sehr oft“.	In the following, you will be asked questions about the last rest period. We ask you to indicate to what extent the following statements about your experience during this time agree with each other. You can give the following ratings: "not at all", "almost not", "a little", "often" and "very often".
1	Ich dachte immer wieder an meine Probleme.	I kept thinking about my problems.
2	Ich verharrte im Denken an Dinge, die mich beunruhigen.	I persisted in thinking about things that worried me.
3	Meine Gedanken wiederholten sich, ohne dass ich zu einer Lösung kam.	My thoughts repeated themselves without me coming to a solution.
4	Ich verlor mich in meinen negativen Gedanken.	I lost myself in my negative thoughts.
5	Ich konnte meine Gedanken nur mühsam festhalten.	I could only hold my thoughts with difficulty.
6	Ich konnte mich nicht von meinen negativen Gedanken lösen.	I could not get away from my negative thoughts.
7	Ich war bei der Sache.	My mind was on the matter at hand.
8	Ich dachte darüber nach, warum ich mich in bestimmten Situationen falsch verhalten habe.	I thought about why I was behaving wrongly in certain situations.
9	Ich fragte mich, warum ich Probleme habe, die andere nicht haben.	I wondered why I had problems that others didn't.
10	Ich fragte mich, womit ich meine momentane Lebenssituation verdient habe.	I asked myself what I did to deserve my current situation in life.
11	Ich dachte darüber nach, warum ich die Dinge nicht besser in den Griff bekomme.	I thought about why I can't get a better handle on things.
12	Ich dachte an all meine Defizite und Misserfolge, Macken und Fehler.	I thought about all my shortcomings and failures, quirks and mistakes.
13	Ich konnte flexibel zwischen meinen Gedanken hin und her schalten.	I had the flexibility to switch back and forth between my thoughts.
14	Ich dachte an vergangene Situationen, die ich bereue.	I thought of past situations that I regret.
15	Ich machte mir Selbstvorwürfe.	I blamed myself.
16	Ich verlor mich in Gedanken an Vergangenes.	I got lost in thoughts of things past.
17	Ich war von meinen Problemen und Sorgen stark vereinnahmt.	I was strongly absorbed by my problems and worries.
18	Meine negativen Gedanken ließen mich nicht los.	My negative thoughts did not let me go.

Supplementary material S5: Analysis using reliable change indices in order to define category change

Predicting change in RRS-categories.

Time. Fitting our first model using only the number of days between T_1 and T_{lab}as a predictor, we found that time between measurements did not predict changing RRS-categories (β = 0.008, z = 1.042, p = .298).

SPSS Anomaly Index. Next, we fitted three models each including the Anomaly Case Index generated by SPSS for the respective RRS-assessment (T_1 vs. T_2 vs. T_{lab}). While the Anomaly Index of T_1 (β = -0.914, z = -0.851, p = .395) and the Anomaly Index of T_{lab} did not yield significant predictors (β = 0.553, z = 0.578, p = .563), the Anomaly Index of T_2 (β = 2.855, z = 1.955, p = .051) yielded marginal significance.

Careless package. Next, we used indexes of the careless package. Entering the corresponding indices of the respective RRS-assessments (T_1 vs. T_2 vs. T_{lab}) separately and as an interaction with each other did not yield any significant predictor. When fitting logistic regression models using the Mahalanobis distances of the respective RRS-assessment (T_1 vs. T_2 vs. T_{lab}), we did not observe any significant predictors (all p´s > 0.546).

SoSci Survey response time indexes. Lastly, we fitted logistic regression models using the time indexes of SoSci-Survey (consequently they were only available for the online assessments at $T_1$and T_2). Again, we entered each predictor on their own and as an interaction of both predictors, however we observed no significant effects (all p´s > 0.208).

Baseline state rumination at T_{lab}. In order to investigate a potential bias in RRS-ratings due to a confound with current state rumination, we lastly fitted logistic regression models using the interaction of RRS-scores at T_1 vs. T_2 vs. T_{lab} with baseline state rumination ratings at T_{lab}. As a result, we did not observe any significant or marginally significant predictors (all p´s > 0.140).

Effectiveness of the changes made in the recruitment procedure. Lastly, we investigated whether any category change between T_1 and T_{lab} was significantly predicted by the time (prior to vs. after changing the recruitment procedure). This resulted again in a non-significant effect (β = -0.740, z = -1.369, p = .171).

Crosstables of the absolute and relative frequency participants changing and not changing categories were flagged by the respective algorithm.

			Category change		
Index	Identification rate		no	yes	total
SPSS Anomaly Index T_1	not flagged	count	77	27	104
		% within SPSS Anomaly Index T_1	74,0%	26,0%	100,0%
		% within category change	100,0%	100,0%	100,0%
		% of total	74,0%	26,0%	100,0%
	flagged	count	0	0	0
		% within SPSS Anomaly Index T_1	0,0 %	0,0 %	0,0 %
		% within category change	0,0 %	0,0 %	0,0 %
		% of total	0,0 %	0,0 %	0,0 %
	total	count	77	27	104
		% within SPSS Anomaly Index T_1	74,0%	26,0%	100,0%
		% within category change	100,0%	100,0%	100,0%
		% of total	74,0%	26,0%	100,0%
SPSS Anomaly Index T_2	not flagged	count	46	13	59
		% within SPSS Anomaly Index T_2	78,0%	22,0%	100,0%
		% within category change	100,0%	92,9%	98,3%
		% of total	76,7%	21,7%	98,3%
	flagged	count	0	1	1
		% within SPSS Anomaly Index T_2	0,0%	100,0%	100,0%
		% within category change	0,0%	7,1%	1,7%
		% of total	0,0%	1,7%	1,7%
	total	count	46	14	60
		% within SPSS Anomaly Index T_2	76,7%	23,3%	100,0%
		% within category change	100,0%	100,0%	100,0%
		% of total	76,7%	23,3%	100,0%
SPSS Anomaly Index T_{lab}	not flagged	count	72	24	96
		% within SPSS Anomaly Index T_{lab}	75,0%	25,0%	100,0%

		% within category change	100,0%	100,0%	100,0%
		% of total	75,0%	25,0%	100,0%
	flagged	count	0	0	0
		% within SPSS Anomaly Index T_{lab}	0,0 %	0,0 %	0,0 %
		% within category change	0,0 %	0,0 %	0,0 %
		% of total	0,0 %	0,0 %	0,0 %
	total	count	72	24	96
		% within SPSS Anomaly Index T_{lab}	75,0%	25,0%	100,0%
		% within category change	100,0%	100,0%	100,0%
		% of total	75,0%	25,0%	100,0%
MAD T_1	not flagged	count	57	24	81
		% within MAD T_1	70,4%	29,6%	100,0%
		% within category change	82,6%	96,0%	86,2%
		% of total	60,6%	25,5%	86,2%
	flagged	count	12	1	13
		% within MAD T_1	92,3%	7,7%	100,0%
		% within category change	17,4%	4,0%	13,8%
		% of total	12,8%	1,1%	13,8%
	total	count	69	25	94
		% within MAD T_1	73,4%	26,6%	100,0%
		% within category change	100,0%	100,0%	100,0%
		% of total	73,4%	26,6%	100,0%
MAD T_2	not flagged	count	37	12	49
		% within MAD T_2	75,5%	24,5%	100,0%
		% within category change	94,9%	85,7%	92,5%
		% of total	69,8%	22,6%	92,5%
	flagged	count	2	2	4
		% within MAD T_2	50,0%	50,0%	100,0%

		% within category change	5,1%	14,3%	7,5%
		% of total	3,8%	3,8%	7,5%
	total	count	39	14	53
		% within MAD T_2	73,6%	26,4%	100,0%
		% within category change	100,0%	100,0%	100,0%
		% of total	73,6%	26,4%	100,0%
MAD T_{lab}	not flagged	count	54	20	74
		% within MAD T_{lab}	73,0%	27,0%	100,0%
		% within category change	79,4%	90,9%	82,2%
		% of total	60,0%	22,2%	82,2%
	flagged	count	14	2	16
		% within MAD T_{lab}	87,5%	12,5%	100,0%
		% within category change	20,6%	9,1%	17,8%
		% of total	15,6%	2,2%	17,8%
	total	count	68	22	90
		% within MAD T_{lab}	75,6%	24,4%	100,0%
		% within category change	100,0%	100,0%	100,0%
		% of total	75,6%	24,4%	100,0%
Time_Deg T_1	not flagged	count	60	19	79
		% within Time_Deg T_1	75,9%	24,1%	100,0%
		% within category change	87,0%	76,0%	84,0%
		% of total	63,8%	20,2%	84,0%
	flagged	count	9	6	15
		% within Time_Deg T_1	60,0%	40,0%	100,0%
		% within category change	13,0%	24,0%	16,0%
		% of total	9,6%	6,4%	16,0%
	total	count	69	25	94
		% within Time_Deg T_1	73,4%	26,6%	100,0%

		% within category change	100,0%	100,0%	100,0%
		% of total	73,4%	26,6%	100,0%
Time_Deg T_2	not flagged	count	35	14	49
		% within Time_Deg T_2	71,4%	28,6%	100,0%
		% within category change	89,7%	100,0%	92,5%
		% of total	66,0%	26,4%	92,5%
	flagged	count	4	0	4
		% within Time_Deg T_2	100,0%	0,0%	100,0%
		% within category change	10,3%	0,0%	7,5%
		% of total	7,5%	0,0%	7,5%
	total	count	39	14	53
		% within Time_Deg T_2	73,6%	26,4%	100,0%
		% within category change	100,0%	100,0%	100,0%
		% of total	73,6%	26,4%	100,0%
RSI T_1	not flagged	count	68	25	93
		% within RSI T_1	73,1%	26,9%	100,0%
		% within category change	98,6%	100,0%	98,9%
		% of total	72,3%	26,6%	98,9%
	flagged	count	1	0	1
		% within RSI T_1	100,0%	0,0%	100,0%
		% within category change	1,4%	0,0%	1,1%
		% of total	1,1%	0,0%	1,1%
	total	count	69	25	94
		% within RSI T_1	73,4%	26,6%	100,0%
		% within category change	100,0%	100,0%	100,0%
		% of total	73,4%	26,6%	100,0%
RSI T_2	not flagged	count	38	14	52
		% within RSI T_2	73,1%	26,9%	100,0%

		% within category change	97,4%	100,0%	98,1%
		% of total	71,7%	26,4%	98,1%
	flagged	count	1	0	1
		% within RSI T_2	100,0%	0,0%	100,0%
		% within category change	2,6%	0,0%	1,9%
		% of total	1,9%	0,0%	1,9%
	total	count	39	14	53
		% within RSI T_2	73,6%	26,4%	100,0%
		% within category change	100,0%	100,0%	100,0%
		% of total	73,6%	26,4%	100,0%

Note. MAD = Mahalanobis distance ($p < .05$); Time_Deg = Index implemented in SoSci Survey penalting fast response times; RSI = Relative Speed Index (Index implemented in SoSci Survey).

Supplementary material S6: ICCs dependent on the sample

Sample S1	type	ICC	F	df1	df2	p-value	CI
single_raters_absolute	ICC1	0.75	6.9	40	41	< .001	[0.57; 0.86]
single_random_raters	ICC2	0.75	9.3	40	40	< .001	[0.47; 0.88]
single_fixed_raters	ICC3	0.80	9.3	40	40	< .001	[0.66; 0.89]
average_raters_absolute	ICC1k	0.85	6.9	40	41	< .001	[0.73; 0.92]
average_random_raters	ICC2k	0.86	9.3	40	40	< .001	[0.64; 0.94]
average_fixed_raters	ICC3k	0.89	9.3	40	40	< .001	[0.80; 0.94]

Sample S2	type	ICC	F	df1	df2	p-value	CI
single_raters_absolute	ICC1	0.88	22	52	106	< .001	[0.81; 0.92]
single_random_raters	ICC2	0.88	23	52	104	< .001	[0.81; 0.92]
single_fixed_raters	ICC3	0.88	23	52	104	< .001	[0.82; 0.92]
average_raters_absolute	ICC1k	0.95	22	52	106	< .001	[0.93; 0.97]
average_random_raters	ICC2k	0.95	23	52	104	< .001	[0.93; 0.97]
average_fixed_raters	ICC3k	0.95	23	52	104	< .001	[0.93; 0.97]

Sample S3	type	ICC	F	df1	df2	p-value	CI
single_raters_absolute	ICC1	0.91	32	81	164	< .001	[0.88; 0.94]
single_random_raters	ICC2	0.91	34	8	162	< .001	[0.87; 0.94]
single_fixed_raters	ICC3	0.92	34	81	162	< .001	[0.88; 0.94]
average_raters_absolute	ICC1k	0.97	32	81	164	< .001	[0.95; 0.98]
average_random_raters	ICC2k	0.97	34	81	162	< .001	[0.95; 0.98]
average_fixed_raters	ICC3k	0.97	34	81	162	< .001	[0.96; 0.98]
